# Cortical activity upon awakening from sleep reveals consistent spatio-temporal gradients across sleep stages in human EEG

**DOI:** 10.1016/j.cub.2025.06.064

**Published:** 2025-08-18

**Authors:** Aurélie M. Stephan, Jacinthe Cataldi, Amrita Singh Virk, Francesca Siclari

**Affiliations:** 1 Center for Investigation and Research on Sleep, Lausanne University Hospital, 1011 Lausanne, Switzerland; 2 The Sense Innovation and Research Center, 1007 Lausanne and Sion, Switzerland; 3 The Netherlands Institute for Neuroscience, 1105 BA Amsterdam, the Netherlands

**Keywords:** awakening, arousal, sleepiness, sleep inertia, high-density EEG, sleep-to-wake transition, sleep slow waves, wake-like activity, sleep, consciousness

## Abstract

How does the brain awaken from sleep? Several studies have suggested that the awakening process occurs asynchronously across brain regions, but the precise nature of these changes and how they are reflected in human electroencephalography (EEG) remains unknown. Here, we recorded 1,073 awakenings and arousals with high-density EEG and mapped brain activity at a second-to-second timescale around movement onset using source modeling. We found that cortical activity upon awakening progressed along highly consistent spatial and frequency gradients. In awakenings and arousals from non-rapid eye movement (NREM) sleep, transient increases in low-frequency power preceded increases in high-frequency power by a few seconds, whereas awakenings from REM sleep were mainly characterized by increases in high-frequency power. Regardless of sleep stage, high-frequency changes were first seen in frontal and last in occipital and inferior-temporal cortical areas, whereas low-frequency changes in NREM sleep started in a centro-parietal “hotspot,” progressed frontally, and reached occipital and inferior-temporal regions last. Finally, the presence of these spatio-temporal arousal patterns during sleep, before participants were awakened by sounds, was followed by lower sleepiness ratings upon awakening. These results indicate a consistent spatio-temporal EEG signature of the awakening process that likely reflects the structural organization of arousal systems. Importantly, a transient increase in slow EEG frequencies, which are normally associated with sleep, is inherent to the arousal process and functionally correlates with feeling more awake when awakening from NREM sleep. These findings have important implications for the interpretation of arousal signals and the detection of incomplete awakenings in sleep disorders.

## Highlights

• High-density EEG reveals consistent cortical activation patterns upon awakening
• High-frequency arousal changes follow a front-to-back gradient across sleep stages
• Low-frequency arousal changes in NREM sleep start in a posterior-medial hotspot
• Different slow wave types show opposite relation to sleepiness upon awakening

## Graphical abstract

**Figure:**
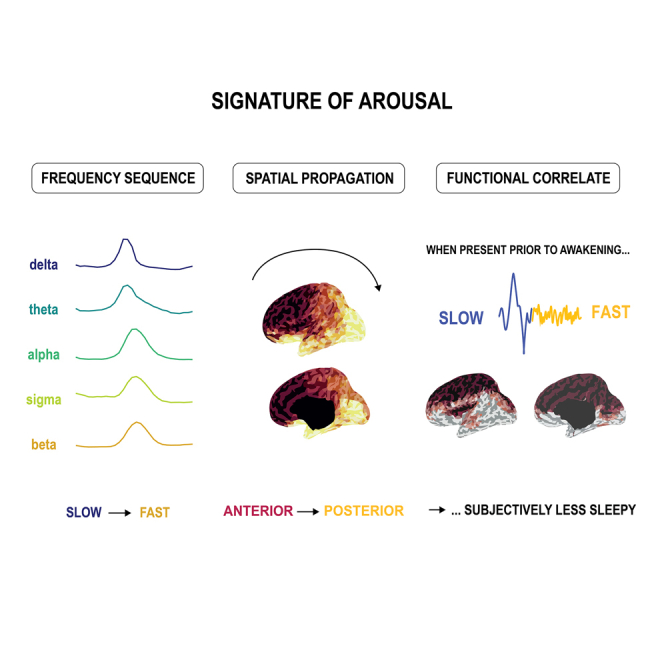

## Teaser

By analyzing over a thousand awakenings, Stephan et al. identify a consistent EEG signature of the awakening process in the human brain. This signature unfolds in distinct temporal and spatial sequences, varies with sleep stage, and is linked to subjective alertness, offering new insight into the neural dynamics of arousal.

## Introduction

The awakening from sleep encompasses a series of remarkable changes. Within a short time, individuals regain waking consciousness, reorient themselves in time, and reconnect with their surroundings, becoming ready to interact with the world again. How does the brain accomplish this transition safely and efficiently? Several behavioral and imaging studies have suggested that the awakening process is not an all-or-none phenomenon but, instead, heterogeneous in space and time, meaning that it occurs asynchronously across different brain regions.^1^ An early positron emission tomography (PET) study, for instance, showed that when transitioning from sleep to wakefulness, blood flow reaches waking levels most quickly in centroencephalic regions, including the brainstem and thalamus, followed by most cortical regions only minutes later.^2^ A recent study using fast functional magnetic resonance imaging (fMRI) at ultra-high field (7 Tesla)^3^ investigated changes at a smaller temporal scale, when participants activated a button press upon awakening from sleep. It documented an orderly sequence of cerebral activations and deactivations, starting before movement onset in the thalamus and cingulate cortices and followed, several seconds later, by the remainder of cortical regions. Similar sequential activations in thalamic and cortical regions were documented in another recent fMRI study.^4^ However, overall, these imaging techniques do not allow one to draw inferences on neural dynamics at a smaller timescale and are not suited to record sleep in natural physiological conditions. Intracranial recordings, with their ability to record activity from restricted neural populations, have provided valuable insight into the local nature and the spatial heterogeneity of sleep and (micro)arousals.^5^^,^^6^^,^^7^ They are, however, restricted to specific patient populations and cover limited regions within a given patient. Conventional electroencephalography (EEG), on the other hand, is the standard technique to record sleep in a naturalistic setting and across clinical disorders but, in its usual setup, uses only a few electrodes. Previous studies have shown that, compared with the pre-sleep period, EEG recordings obtained immediately after the awakening display higher low-frequency spectral power typical of sleep,^8^ especially in posterior cortical regions,^9^^,^^10^ or a higher low-to-high-frequency power ratio.^11^ These changes typically revert over several minutes, in parallel to the reestablishment of functional connectivity patterns of wakefulness^11^^,^^12^ and cognitive performance.^13^^,^^14^ Thus, although it is well known that, overall, the EEG upon awakening becomes progressively more “wake-like,” regional EEG changes at the precise moment of the transition from sleep to wakefulness have not been investigated in detail.

Documenting a spatio-temporal EEG signature of the awakening process is, however, of major interest, not only to better understand how the regional reestablishment of wakefulness affects cognition and behavior^15^^,^^16^ but also because many sleep disorders,^17^^,^^18^ including insomnia and parasomnias,^19^^,^^20^^,^^21^^,^^22^^,^^23^^,^^24^ are characterized by incomplete, excessive, or abnormally timed arousals. Better understanding the spatial dynamics underlying these arousals may thus improve their detection and help identify the underlying neural substrates. In addition, animal studies have made considerable progress in dissecting the circuits underlying arousal processes and associated EEG signatures.^25^^,^^26^^,^^27^^,^^28^^,^^29^^,^^30^^,^^31^ Applying these insights to human EEG recordings allows one to infer possible neural mechanisms underlying the awakening process.

Therefore, in this study, we took advantage of high-density EEG with 256 channels and source modeling analyses to map regional brain activity at a second-to-second timescale when individuals start to move upon awakening. To investigate the awakening process in naturalistic conditions, we first analyzed spontaneous awakenings (111 awakenings across 20 individuals) that occurred across different times of the night and sleep stages. To relate these EEG changes to a functional measure of arousal, we then analyzed awakenings that were provoked by an alarm sound (572 awakenings across 20 individuals) on different nights, after which the same participants gave subjective ratings of their sleepiness.

With this approach, we were able to delineate an EEG signature of the awakening process that progressed along a consistent spatial gradient, from fronto-central to occipital areas. Awakenings from non-rapid eye movement (NREM) sleep also displayed a consistent frequency gradient, with a low-frequency component preceding a high-frequency component by a few seconds, mirroring the timing of previously documented thalamo-cingulate and cortical activations, respectively. Drawing on recent findings in rodents, we discuss how the degree of cortical bistability and activation patterns of arousal systems may affect the expression of arousal at the EEG level and account for our results.

## Results

### The temporal dynamics of the awakening process

#### Temporal dynamics of spontaneous awakenings

**Figure 1:**
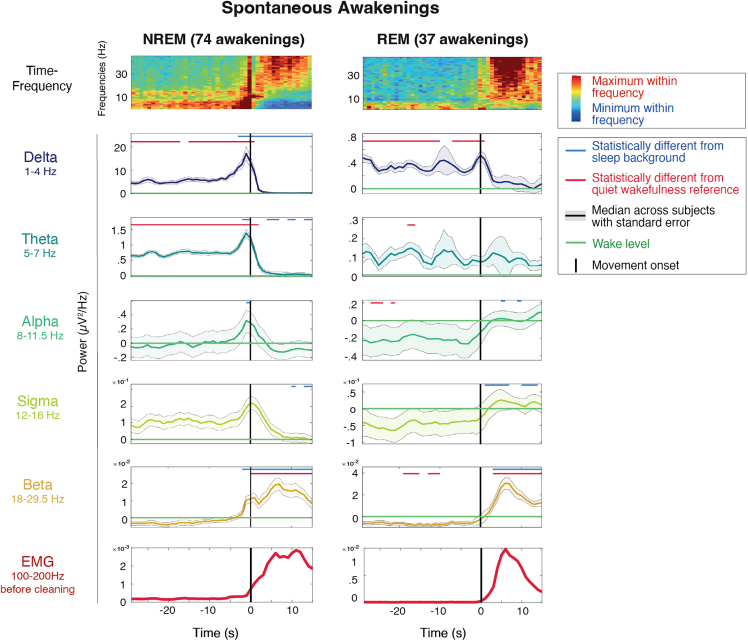
Time course of EEG activity during spontaneous awakenings from NREM and REM sleep Time course of fast Fourier transform across frequencies (first row) and separated into six frequency bands of interest (second to bottom row) averaged across the 185 innermost channels for NREM sleep (left, *N* = 19 participants) and REM sleep (right, *N* = 17 participants). The time-frequency plots (first row) are logged and *Z* scored across time for each subject, and the median across subjects is plotted. The frequency band plots (second to bottom row) are normalized on quiet wakefulness for each subject, the median across subjects is plotted in bold and the standard error as an area around it. The black line at 0 denotes movement onset. The green horizontal line displays the quiet wakefulness reference. On the top of each frequency band plot, the blue line indicates the time points at which the power is significantly different from the background sleep reference (−45 to −30 s) and the red line indicates the time points at which the power is significantly different from the pre-sleep quiet wakefulness reference. See also Figures S1–S4.

Awakenings out of sleep were identified visually, according to international scoring criteria.^32^ Only awakenings lasting longer than 30 s and including clear-cut movement activity were included (see STAR Methods section for details). To visualize global EEG changes associated with the awakening process and assess the validity of our data, we first computed the time-frequency dynamics of the fast Fourier transform across channels on spontaneous awakenings, time-locked to movement onset (Figure 1, first row). As expected, spontaneous awakenings out of NREM sleep were characterized by a transition from a low-frequency (<8 Hz) and sigma (12–15 Hz) power dominance, reflecting the presence of sleep slow waves and spindles, respectively, to a high-frequency (>15 Hz) and alpha power dominance (8–10 Hz) typical of wakefulness. Around movement onset, a short-lived power increase affecting all frequency bands was observed. Spontaneous awakenings out of REM sleep also displayed a shift from a low-frequency (<10 Hz) to a high-frequency (>10 Hz) activity dominance.

We then decomposed the time course of the fast Fourier transform into different frequency bands (Figures 1 rows 2–6 and S1). This analysis confirmed that most frequencies in NREM sleep peaked around movement onset, reaching levels that were significantly higher than both prior sleep and resting wakefulness. Interestingly, we observed a temporal lag of the peak activity across frequencies, with lower frequencies (<7 Hz) peaking first (with a median of −1 s relative to movement onset), followed sequentially by alpha (0 s) and beta power (+6 s). Analyzing N2 and N3 awakenings separately revealed very similar temporal dynamics (Figure S2). In spontaneous REM sleep awakenings, a well-defined increase above sleep and waking levels was identified only for beta power (at +8 s).

Next, to determine whether the frequency gradient in spontaneous awakenings was present not only in the trial average but also in individual trials, we evaluated each trial for the presence of a peak in activity above baseline levels in the delta and beta bands (as representative for low and high EEG frequencies, see STAR Methods section for details). The analysis confirmed that the large majority of awakenings in NREM sleep (69 of 74 trials, 93%) displayed a peak in both delta and beta power, with the delta peak preceding the beta peak in 91% of cases (63 trials) (Figures S3A and S3B). Visual inspection and time-frequency analysis of individual trials also confirmed that the frequency changes were visible within single trials and that they were consistent between trials (Figure S3C) and revealed that the delta peak frequently represented a K-complex.

#### Temporal dynamics of spontaneous arousals

**Figure 2:**
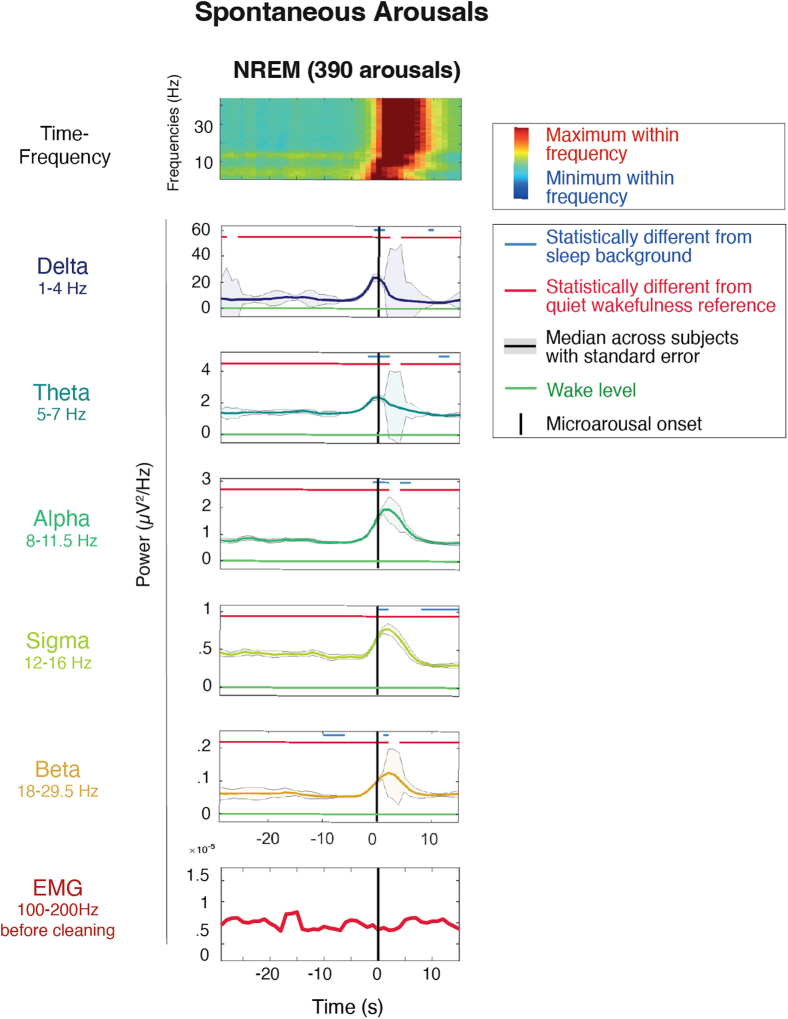
Time course of EEG activity during spontaneous arousals without EMG activation in NREM sleep Arousals were visually scored and lasted at least 3 s and less than 15 s. Time course of fast Fourier transform across frequencies (first row) and separated into six frequency bands of interest (second to bottom row) averaged across the 185 innermost channels in NREM sleep (*N* = 15 participants). The time-frequency plots (first row) are logged and *Z* scored across time for each subject, and the median across subjects is plotted. The frequency band plots (second to bottom row) are normalized on quiet wakefulness for each subject, the median across subjects is plotted in bold and the standard error as an area around it. The black line at 0 denotes EEG arousal onset. The green horizontal line displays the quiet wakefulness reference. On the top of each frequency band plot, the blue line indicates the time points at which the power is significantly different from the background sleep reference (−45 to −30 s) and the red line indicates the time points at which the power is significantly different from the pre-sleep quiet wakefulness reference.

We then performed the same analyses on NREM arousals, which represent micro-awakenings lasting at least 10 s and occur against a background of stable sleep. Arousals were scored according to standard criteria^33^ as “abrupt shifts of EEG frequency including alpha, theta, and/or frequencies greater than 16 Hz (but not spindles) that last at least 10 s.” To disentangle potential movement-related activity from brain activity, here we specifically focused on arousals without electromyographic (EMG) activation. This analysis, centered on arousal onset, revealed similar spectral power dynamics to full awakenings (Figure 2), including a distinct peak in low-frequency power followed by a high-frequency power peak (>15 Hz), with similar temporal delays to spontaneous awakenings (Figure 2). Thus, the temporal spectral power dynamics associated with the awakening process are also present in incomplete awakenings (arousals) and are unlikely to represent movement artifacts because they were present in arousals without EMG activations.

### The spatial dynamics of the awakening process

We next investigated whether the spectral power peaks observed in the different frequency bands upon awakening exhibited a consistent topographical pattern. At the moment of the power peak observed in the channel average, power was significantly higher with respect to baseline sleep and quiet wakefulness levels across almost all electrodes and frequency bands, except for alpha power in occipital regions in NREM sleep, which did not significantly change (Figure S4). This finding suggests that the spectral power increase associated with the awakening is widespread, consistently affecting nearly all brain areas. We then examined whether spectral power peaks across different frequency bands followed a consistent spatial sequence. To this aim, for each frequency band, we ranked the voxels in the order of the detected peaks (see STAR Methods section for details).

**Figure 3:**
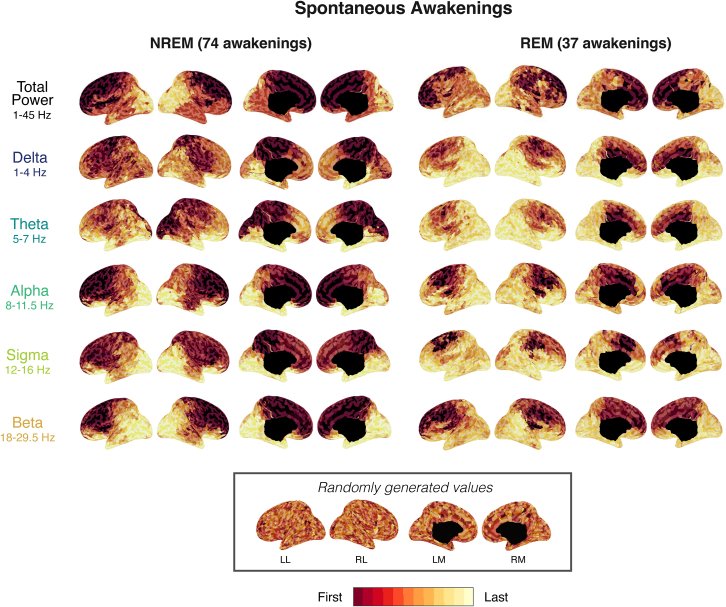
Cortical maps of latencies to peak activity for spontaneous awakenings The timing of peak activity is displayed on source-reconstructed data for NREM awakenings (*N* = 19 participants) and REM awakenings (*N* = 17 participants). Latency maps display, for each frequency band, the timing at which regions reach their maximal activity in the window from −15 to +15 s around movement onset. Red indicates regions peaking early and yellow indicates regions peaking late. For comparison, a cortical map of randomly generated values is shown at the bottom. LL, left lateral; LM, left medial; RL, right lateral; and RM, right medial. See also Figures S4 and S5 and Table S1.

In spontaneous awakenings from NREM and REM sleep, global power (1–45 Hz) peaked following a clear-cut anterior-to-posterior gradient—first in anterior brain regions and last in posterior brain regions (Figure 3, first row; for a list of absolute latencies; see Table S1). The cortical regions in which global power consistently peaked first across behavioral states (REM/NREM) were prefrontal regions, including the anterior cingulate cortex and the superior, medial, and precentral gyri, as well as the insula (see supplemental information for full voxel rankings). In NREM sleep, central regions—including the motor cortex, ventral and posterior cingulate cortex, and parietal lobule—were also among the first to reach peak spectral power. Voxels peaking last, across both states and conditions, were primarily located in posterior cortical areas (cuneus, precuneus, lingual gyrus, and inferior, middle, and superior occipital gyri, as well as in the posterior cingulate cortex, supramarginal gyrus, and angular gyrus).

Analysis of latency of peaks revealed that the global, anterior/central to posterior latency gradient was present across frequency bands (Figure 3, rows 2–6) and behavioral states (REM/NREM). However, some frequency-specific regional differences also emerged. Specifically, for delta and theta power in NREM sleep, spectral power peaks first appeared in central and medial posterior regions, then progressed toward frontal areas, and reached lateral and inferior occipital regions last.

Importantly, analyzing spatial frequency propagation during an arbitrary time window of consolidated sleep did not reveal consistent propagation patterns, suggesting that the spatial gradient shown in Figure 3 is locked to the awakening process (Figure S5).

### Subjective sleepiness

To assess the relationship between subjective sleepiness and the awakening process, we analyzed awakenings induced by an alarm in the same participants on different nights, after which subjective ratings of sleepiness were collected. Overall, these provoked awakenings displayed comparable spectral frequency changes (Figure S6A) and spatial dynamics (Figure S6B) over time, with the exception of REM sleep awakenings, which, unlike spontaneous awakenings, also showed a power peak in the delta range. Provoked NREM awakenings showed a less pronounced delta peak than spontaneous awakenings, possibly because they occurred in periods of already elevated slow wave activity.

#### Subjective sleepiness across sleep stages

**Figure 4:**
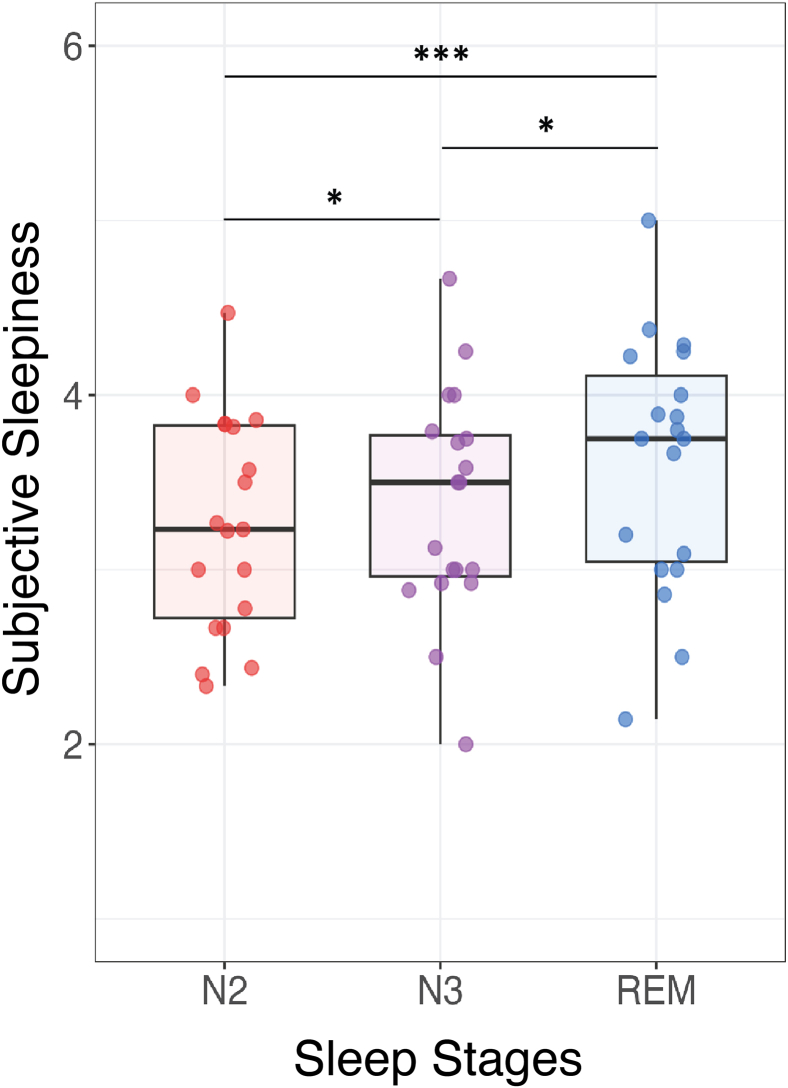
Reported sleepiness upon awakening from N2, N3, and REM sleep Subjective sleepiness (from 1 = not sleepy at all to 5 = very sleepy) as a function of sleep stage (N2, N3, REM; *N* = 20 participants). Each point represents an individual, the box displays the 25th to 75th percentile of data, and the vertical bars represent the confidence interval. Post hoc comparisons of significant main and interaction effects, evaluated using generalized linear mixed models and corrected for multiple comparisons, are shown with connecting lines. Asterisks indicate the level of significance: ^∗^*p* < 0.05, ^∗∗^*p* < 0.01, ^∗∗∗^*p* < 0.001.

Participants reported greater sleepiness when awakened from REM sleep compared with NREM sleep (main effect of stage, χ^2^(1) = 17.8, *p* < 0.0001), even when considering N2 and N3 separately (N2-REM: estimate = −0.4, t = −4.1, *p* ≤ 0.0001; N3-REM: estimate = −0.2, t = −1.97, *p* = 0.049; N2-N3: estimate = −0.2, t = −2.2, *p* = 0.02) (Figure 4). After correcting for time of night, sleepiness in N3 and REM was comparable and higher than in N2 sleep (N2-REM: estimate = −0.4, t = −4.2, *p* ≤ 0.0001; N3-REM: estimate = −0.07, t = −0.07, *p* = 0.5; N2-N3: estimate = −0.32, t = −3.5, *p* = 0.0005).

#### Power spectral density upon awakening and subjective sleepiness

**Figure 5:**
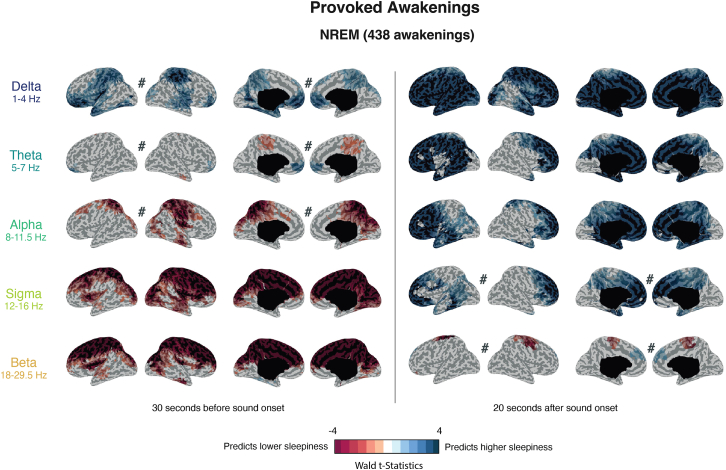
Power spectral density in provoked NREM awakenings predicts subjective sleepiness Results of linear mixed models examining the relationship between sleepiness and power spectral density during NREM awakenings (*N* = 20 participants). Power spectral density was averaged within two time frames around sound onset (30 s before sound onset and 20 s after). For each time frame, maps show power spectral density effects for left and right lateral (LL and RL) and medial cortical regions (LM and RM). The effect of each frequency band on sleepiness was evaluated in separate models. All models included subject identity and time of night as random factors. Wald statistic values (calculated as the squared ratio of the fixed factor estimate over its standard error) are shown at the source level. Voxels with non-significant effects are shown in gray. Contrasts that did not survive the cluster-based correction are indicated with a hash (#) symbol. See also Figure S6.

Participants reported significantly higher sleepiness upon awakening when high-frequency power (above the alpha range) was low prior to movement onset and when low-frequency power (delta through alpha) was high immediately after movement onset in NREM sleep. These correlates were spatially diffuse but tended to spare regions of the occipital and temporal cortices (Figure 5). EEG activity during REM sleep awakenings did not correlate significantly with subjective sleepiness, either before or after movement onset.

**Figure 6:**
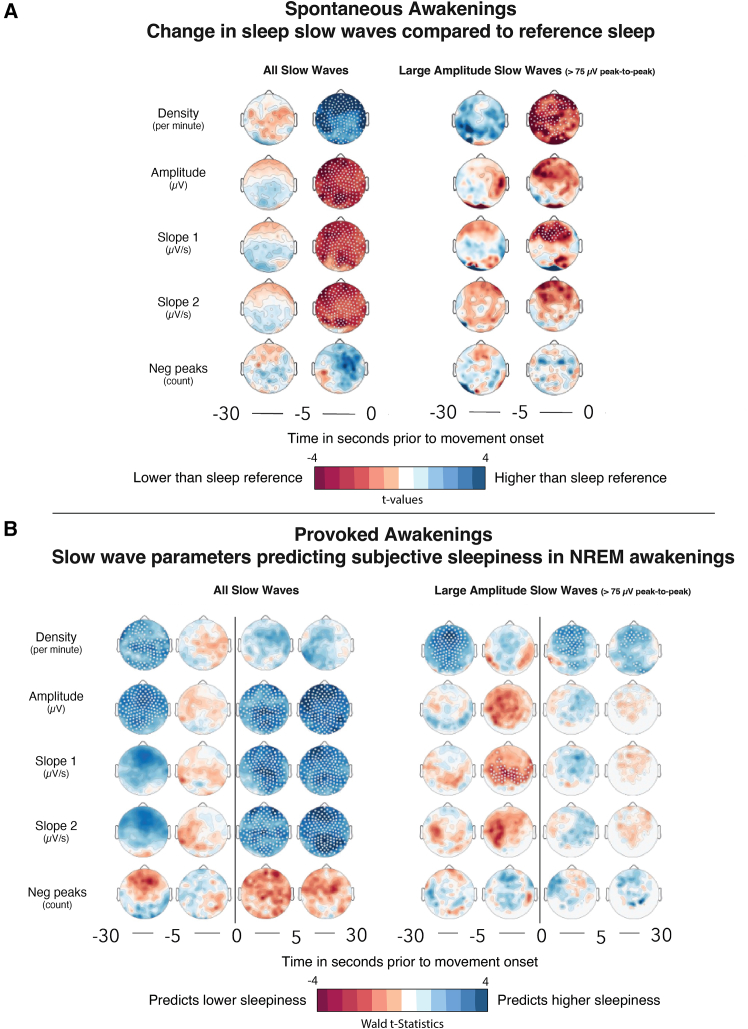
Sleep slow waves during spontaneous awakening and subjective sleepiness (A) Comparing slow wave parameters in the pre-awakening period (−5 to 0 s before movement onset) and background sleep (−30 to −5 s) to a reference window (−45 to −30 s) in spontaneous awakenings from NREM sleep (*N* = 19). Topographical maps of *t* values are shown for all slow waves (left) and those exceeding peak-to-peak amplitudes of 75 μV (right). Red indicates values significantly higher than in the sleep reference window and blue indicates values significantly lower than in the sleep reference window (two-tailed paired t test). (B) Results of linear mixed models explaining the relationship between subjective sleepiness and slow wave parameters for all slow waves (left) and those exceeding peak-to-peak amplitudes of 75 μV (right) for induced awakenings from NREM sleep (*N* = 20). Blue indicates a positive association with subjective sleepiness. All statistical analyses were performed within subject, on a channel-by-channel basis, and were corrected for multiple comparisons using a cluster-based correction. White dots indicate channels for which the statistical comparison was significant after correction. Mixed models included subject and time of night as random factors.

In light of previous work documenting two types of slow waves with opposing subjective correlates, we performed a slow wave analysis with and without applying a minimal amplitude threshold. This approach previously allowed us to dissociate large-amplitude slow waves (representing so-called type I slow waves, including K-complexes), which are likely related to arousal systems, from smaller type II slow waves, primarily composing delta waves.^1^^,^^20^^,^^34^^,^^35^ We first analyzed slow waves associated with spontaneous awakenings, centered on movement onset. Compared with a baseline sleep reference window (−45 to −30 s before movement onset), slow wave parameters remained stable until 5 s before movement onset. Then, immediately before movement onset, slow waves became significantly larger and steeper (Figure 6A, left). Notably, when selecting only large-amplitude slow waves (representative of type I slow waves), we found that they were more numerous immediately before awakening (Figure 6A, right). These findings suggest that the increase in delta power observed prior to the behavioral onset of awakening is attributable to the appearance of very large-amplitude, steep slow waves (type I slow waves). Relating slow wave parameters to subjective sleepiness upon awakening revealed a dissociation between the two types of slow waves. Specifically, the parameters of large-amplitude slow waves (characteristic of type I slow waves) occurring immediately before sound onset were negatively correlated with sleepiness (Figure 6B), whereas the opposite relation was observed when considering all slow waves in the −30 to −5 s window and after movement onset (reflecting mostly type II slow waves). The steeper the type I slow waves immediately before movement onset, and the smaller type II slow waves during baseline sleep and after movement onset, the lower the reported sleepiness.

## Discussion

Our study allowed us to outline an EEG signature of the awakening process both in time and space, across different forms of arousal, and to identify a corresponding functional correlate.

### Spectral power changes: Temporal aspects in NREM sleep

The finding that, in NREM sleep, power increased in all frequency bands upon awakening, including the low-frequency power typical of sleep, is compatible with the fact that full awakenings and arousals are often preceded by a K-complex. This distinctive slow wave can either occur spontaneously or be externally induced by sensory stimulations of various modalities, suggesting a strong link with arousal systems.^36^^,^^37^ Indeed, our slow wave analysis suggests that the slow waves immediately preceding awakenings display the typical hallmarks of K-complexes (type I slow waves). High- and low-frequency power increases have previously been documented during arousal-related events, including arousals and awakenings from NREM sleep,^5^^,^^6^^,^^7^^,^^38^^,^^39^^,^^40^ specific phases of the cyclic alternating pattern in NREM sleep,^40^ respiratory events,^39^ and periodic leg movement events in sleep.^41^ Importantly, intracranial studies have shown that low- and high-frequency increases can occur within the same cortical region.^5^^,^^6^^,^^7^

**Figure 7:**
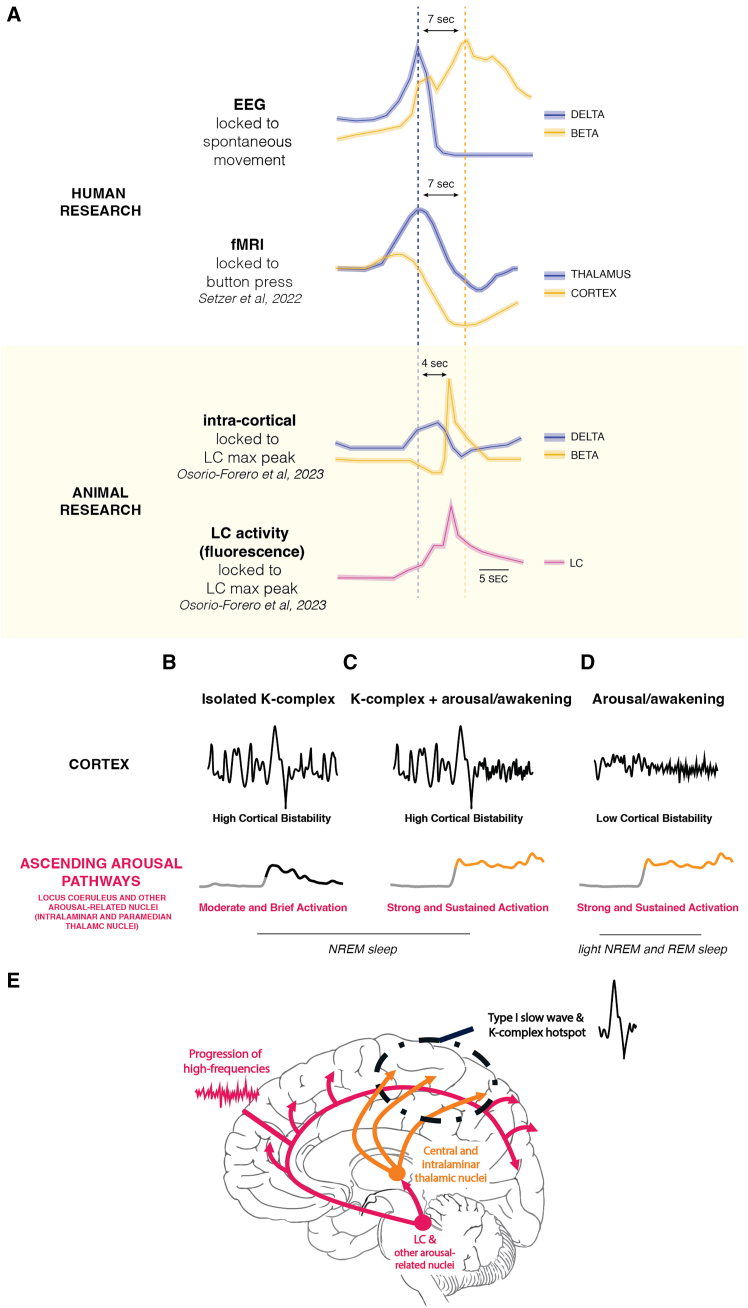
Brain activity changes associated with arousal across recording methods and species and proposed model (A) The sequential peaks in delta and beta power observed upon awakening in the present study (first row) temporally align with various measures reported in previous studies (second to last row). These include (1) thalamic and cortical fMRI blood-oxygen-level-dependent (BOLD) changes upon awakening (second row)—note that the trough in cortical BOLD activity is thought to reflect arousal-induced vasoconstriction associated with increased cortical neural activity— and (2) EEG delta and beta power changes time-locked to peak activity in the LC in mice (third and fourth rows). EEG, electroencephalography; fMRI, functional magnetic resonance imaging; LC, locus coeruleus*.* (B–E) Proposed model: the EEG response during arousal shaped by the interplay between (1) the degree of cortical bistability and (2) the intensity and type of activation of arousal systems (comprising, among other regions, the intralaminar and paramedian thalamic nuclei and the LC). (B) An increase in delta activity in the form of a K-complex is promoted by high cortical bistability, burst firing in arousal-related thalamic nuclei, and weak LC activation. (C) A classical cortical activation (decrease in delta power and increase in high-frequency power) occurs when cortical bistability is low, thalamic nuclei fire tonically, and the LC is strongly activated, either after a K-complex or (C) directly during light NREM or REM sleep (D). Spatially, the initial increase in low-frequency EEG activity in central regions aligns with the hotspot of type I slow waves and to the preferential target of intralaminar thalamic nuclei, whereas the propagation of high-frequency EEG activity reflects the anterior-posterior organization of arousal systems’ cortical projections (E). See text in discussion for explanation and references.

What could this frequency gradient reflect at the neuronal level? Although our study was not designed to address this question, our findings align with recent human fMRI and rodent studies. A fast fMRI study (7T) analyzing awakenings from NREM sleep^3^ identified an initial, fast activation in the thalamus and cingulate cortex just before movement onset—similar to our low-frequency spectral peak—followed by a slower cortical deactivation, likely linked to arousal-induced vasoconstriction, with a similar timing to our high-frequency EEG peak (Figure 7A). A similar two-phase process was observed in other fMRI^4^ and PET studies, though over longer timescales.^2^ Finally, the characteristics of type I slow waves (including K-complexes), which underlie the low-frequency peak, suggest a subcortical origin.^35^ Taken together, these studies support the hypothesis that low-frequency power increases may reflect thalamo-cingulate activation, whereas high-frequency peaks correspond to cortical activation seen in neuroimaging studies.

Furthermore, we hypothesize that the frequency gradient observed in our study directly reflects the activity levels and discharge patterns of arousal-related structures, including the locus coeruleus (LC) and intralaminar thalamic nuclei.^42^^,^^43^ More specifically, the initial low-frequency power increase observed during arousal may primarily reflect the response to a short-lived low-intensity, *subcortical* activation, whereas the high-frequency power increase^42^ could reflect a more sustained activation of subcortical structures. The intensity and duration of these activations likely also determine whether an arousal or a full awakening occurs.^43^ This interpretation is supported by the fact that, in humans, K-complexes, whose presence is reflected in the low-frequency peak, are preferentially evoked by phasic, short-lived, and unexpected stimuli.^44^^,^^45^^,^^46^^,^^47^^,^^48^ In addition, bursting activity in the centromedian nucleus of the thalamus induces cortical slow waves, whereas tonic single-spike activity leads to awakenings.^43^^,^^49^^,^^50^^,^^51^

Other evidence comes from studies examining the activity of the LC. Although its role in inducing full awakenings in both REM and NREM sleep is well established,^52^^,^^53^^,^^54^ more recent studies have demonstrated that the LC exhibits periodic surges in neural population activity even within consolidated NREM sleep, determining windows of cortical and behavioral arousability.^29^^,^^55^ Crucially, when an LC activity surge is present but of moderate amplitude, the cortical EEG (local field power) displays a peak in low frequencies (delta power), whereas at higher LC activity levels, this delta peak is followed, within a few seconds, by an arousal—that is, an increase in high-frequency power and EMG activation—similar to the awakenings and arousals assessed in our study.^42^^,^^43^

### Spectral power changes: Temporal aspects in REM sleep

Unlike spontaneous awakenings out of NREM sleep, those occurring out of REM sleep were not preceded by a low-frequency power peak but were instead associated only with a high-frequency power increase. In NREM sleep, thalamocortical neurons are bistable: they oscillate between two states, each lasting a few hundred milliseconds—a hyperpolarized “down-state” during which neurons are silent (off-period) and a depolarized “up-state” (on-period) characterized by neuronal firing.^56^^,^^57^ When the cortex is bistable, any local activation, spontaneous or externally induced—by sound, electrical, or magnetic stimulation—will eventually provoke a down state and thus induce a slow wave.^58^ The bistability is thought to depend on the dynamics of activity-dependent potassium currents and to be favored by the global neuromodulatory milieu of NREM sleep.^59^^,^^60^ In REM sleep, the neuromodulatory milieu is different: the cortex is not intrinsically bistable,^61^ meaning that a cortical activation is not likely to give rise to a slow wave. The only case in which a slow component was observed in REM awakenings in this study was when they were induced by a loud sound. This suggests that a strong external stimulus may still be able to synchronize activity across thalamocortical neurons even when cortical bistability is low. The variable degree of cortical bistability within NREM sleep could account for the fact that most, but not all, awakenings in NREM sleep were associated with a low-frequency power peak (Figure S4), as also reported previously.^7^^,^^62^ Particularly in light sleep (N1 and early N2), an arousal can indeed directly manifest with cortical activation without an initial low-frequency power increase.^41^^,^^63^

In summary, we hypothesize that the EEG response to the activation of arousal systems depends on two main factors: (1) the intensity and type of activation of subcortical arousal structures (low- vs. high-intensity and short-lived vs. sustained activation) and (2) the bistability of the cortex at the moment of the awakening. Low-intensity, short-lived activations of arousal systems (LC, intralaminar thalamic nuclei) at a moment of high cortical bistability would favor the generation of type I slow waves (including K-complexes) and would thus result in the low-frequency power increase seen in this study, whereas high-intensity, sustained activations of arousal systems in the presence of low cortical bistability would favor a classical EEG activation, consisting of high-frequency power increases and low-frequency power decreases. The latter can be seen in the second part of the NREM awakenings in this study as well as in arousals or awakenings occurring in “light” NREM sleep (stages N1 or early N2) or REM sleep (see Figure 7).

### Spectral power changes: Spatial aspects in NREM and REM sleep

High-frequency EEG changes progressed along an anterior-posterior direction, regardless of behavioral state, suggesting that this gradient reflects a fundamental structural organization of arousal systems.^64^ Studies using other imaging or recording modalities (PET, fMRI, and intracortical EEG recordings) have shown that the brainstem, thalamus, and anterior cingulate cortex are among the first regions to increase their activity upon awakening or arousal,^2^^,^^3^^,^^4^ consistent with our findings. In addition, the distribution of noradrenergic fibers originating in the LC mirrors the EEG changes seen upon awakening: at the level of the cortex, they travel in a cranio-caudal direction, such that a cortical lesion will eliminate the noradrenergic innervation of the cortex caudal to it.^65^ The overall progression of the awakening signature along an anterior-posterior axis is also reminiscent of the cortical traveling of slow waves.^66^

Low-frequency changes in NREM sleep were first seen in medial central (fronto-parietal) regions, before appearing in more frontal regions and lastly in occipital regions. This centro-medial slow wave “hotspot” encompassing the sensorimotor and posterior cingulate cortex corresponds to cortical origins of type I slow waves (including K-complexes)^35^ and to the site where slow waves are preferentially elicited by transcranial magnetic stimulation (TMS).^58^ It is also the region where arousals across sleep show the most consistent spatial changes, as demonstrated by intracranial recordings,^7^ where noradrenaline concentrations peak in human brains^67^ and where noradrenergic fibers are densest across species, followed by frontal and lastly occipital areas.^68^^,^^69^ In addition, these central areas are preferentially targeted by some intralaminar thalamic nuclei,^70^^,^^71^^,^^72^ which produce behavioral and EEG arousal when stimulated in humans and monkeys,^73^^,^^74^^,^^75^ further supporting a link between slow waves and arousal systems.^76^^,^^77^ It is likely that this cortical hotspot for slow wave induction is preferentially recruited via indirect arousal pathways involving the central and intralaminar thalamus, whereas the anterior-posterior gradient seen for high-frequency changes upon awakening could reflect the organization of the direct cortical projections from arousal-related nuclei like the LC (Figure 7E).

### Subjective sleepiness upon awakening

EEG activity correlated with subjective sleepiness upon awakening from NREM sleep but not REM sleep. This lack of correlation during REM sleep may be due to a ceiling effect, as participants generally felt sleepier after REM sleep, resulting in reduced variability in sleepiness ratings. Additionally, the limited number of REM awakenings may have limited statistical power.

During NREM sleep, awakenings preceded by signs of the awakening process (e.g., type I slow waves, fewer delta or type II waves, and high beta power) were linked to lower sleepiness. Less carry-over of slow wave activity into wakefulness also predicted reduced sleepiness, aligning with studies showing high delta power in phase A1 cyclic alternating pattern (CAP) arousals and post-awakening,^78^ when sleep inertia is usually highest.^8^^,^^9^^,^^10^^,^^11^ Similarly, both pre- and post-awakening delta power was shown to correlate with impaired performance.^79^^,^^80^^,^^81^

Crucially, our findings revealed a functional dissociation between two types of slow waves. Although sleepiness positively correlated with type II slow waves during baseline sleep, it negatively correlated with type I slow wave amplitude immediately before movement onset. Slow wave activity is usually expressed in the delta band power and considered a hallmark of sleep, but it encompasses at least two types of distinct oscillations that display opposite characteristics,^35^ relationships to subjective experience,^82^^,^^83^ and developmental trajectories.^84^ This dual nature of slow EEG elements has long been recognized^40^: K-complexes have been described as Janus-faced elements,^85^^,^^86^ promoting either “arousal-inducing” or “sleep-protective” mechanisms.

Here, we suggest that these waveforms, in all cases, reflect the response of a bistable cortex to the phasic activation of subcortical arousal systems. Whether an individual remains asleep, displays an arousal, or undergoes a full awakening will depend on several factors, among which we propose two key ones: (1) intensity and type of activation of subcortical arousal systems (high vs. low and short-lived vs. tonic) and (2) the bistability of the cortex. Testing these scenarios in rodents by selectively manipulating arousal circuits will help verify these hypotheses. This knowledge could then be applied to various clinical conditions, for instance, to identify biomarkers for NREM parasomnias and insomnia subtypes, to predict individual sleepiness in patients with obstructive sleep apnea, or to help control sleep-related seizures.

### Limitations of the study

Several limitations should be considered when interpreting our findings. First, the alignment of EEG data to movement onset was a practical decision, but it may not fully capture variability in the timing of movements related to the arousal process, as movement could potentially occur at varying time points. Additionally, it was not possible to use the same alignment criteria for arousals and full awakenings because only arousals without EMG activation were included in the analysis. Although the study included a large number of awakenings and a participant sample size comparable with previous studies, future research is encouraged to validate these results, using larger and more diverse datasets to ensure that the findings generalize across broader populations. Furthermore, although we used individual EEG coordinates for the source modeling, this process inherently involves estimations that may introduce some uncertainty. Lastly, although subjective sleepiness ratings were collected using a highly standardized protocol with precise instructions and structured interviews, individual differences in self-assessment remain a possible source of variability. Future work may benefit from incorporating additional behavioral measures of sleepiness to complement self-reports.

## Resource availability

### Lead contact

Requests for further information and resources should be directed to, and will be fulfilled by, the lead contact, Francesca Siclari (f.siclari@nin.knaw.nl).

### Materials availability

This study has not generated any new reagents.

### Data and code availability

• EEG data have been deposited on Zenodo at Database: https://doi.org/10.5281/zenodo.15836096 and are publicly available as of the date of publication. This database includes power spectral density and slow wave data associated to the main figures of this publication.
• All original code has been deposited on Zenodo at Database: https://doi.org/10.5281/zenodo.15838327 and is publicly available as of the date of publication. This repository contains all scripts allowing reproduction of the main figures of this publication.
• Any additional information required to reanalyze the data reported in this paper is available from the lead contact upon request.

## Acknowledgments

This work was supported by the Swiss National Science Foundation Ambizione grant PZ00P3_173955 and the ERC grant Dreamscape 101039782 awarded to F.S. The authors thank Anita Lüthi, Alejandro Osorio-Forero, and Eus van Someren for insightful comments and discussions.

## Author contributions

Conceptualization, F.S.; methodology, F.S. (serial awakening paradigm) and A.M.S. (analysis algorithms); software and formal analysis, A.M.S., J.C., and A.S.V.; investigation, J.C.; writing – original draft, F.S. and A.M.S.; writing – review and editing, all authors; visualization, A.M.S.; supervision and project administration, F.S.; and funding acquisition, F.S.

## Declaration of interests

The authors declare no competing interests.

## STAR★Methods

### Key resources table

**Table:** 

REAGENT or RESOURCE	SOURCE	IDENTIFIER
**Deposited data**		

Database	This paper	https://doi.org/10.5281/zenodo.15836096
Script	This paper	https://doi.org/10.5281/zenodo.15838327

**Software and algorithms**		

MATLAB 2022b	RRID: SCR_001622	https://www.mathworks.com/products/matlab.html
R Studio 2024.12.0	RRID: SCR_000432	https://www.rstudio.com/

### Experimental model and subject details

#### Selection of participants

Twenty participants (age 38,3 ± 7,4 years (mean ± SD), range: 25 – 51, 15 female) were recruited through advertisement and word of mouth. They had to have regular bed and rise times and good subjective sleep quality (Pittsburgh Sleep Quality Index^87^ <5). Subjects with extreme chronotypes (Horne and Ostberg morningness-eveningness questionnaire^88^ scores >70 or <30), excessive daytime sleepiness (Epworth Sleepiness Scale^89^ >10 ), suffering from neurological, psychiatric or medical disorders affecting sleep, taking regular medication (besides birth control) or who were pregnant were not included in the study. Written informed consent was obtained for all the participants and the study was approved by the local ethical committee (commission cantonale éthique de la recherche sur l’être humain du canton de Vaud).

#### Experimental procedure

Participants first completed an uninterrupted (baseline) sleep recording, followed by two additional sleep recordings during which they were serially awakened with a computerized alarm sound lasting 1.5 s. They were then interviewed via intercom regarding their sleep-related conscious experiences and subjective sleepiness upon awakening. Awakenings were scheduled during periods of consolidated sleep —approximately 10 min after the first sleep spindle for NREM sleep awakenings —and 10 min after the beginning of REM sleep for REM sleep awakenings. After each awakening, participants were asked to estimate their immediate sleepiness by answering: “How sleepy do you feel right now, on a scale from 1 to 5, – 1 indicating not feeling sleepy at all and 5 feeling extremely sleepy?”. After the interview participants were allowed to return back to sleep. The baseline recording was used to analyze spontaneous awakenings, and the two serial awakening recordings to analyze the provoked awakening and to relate the awakening process to subjective sleepiness ratings.

Recordings started at approximately 11:30 pm and ended at 6:30 am the next morning. Participants were instructed to maintain a regular sleep-wake schedule during the week preceding the recordings, and compliance was verified using wrist-worn actigraphy. EEG was recorded with a 256-channel system (Electrical Geodesics, Inc., Eugene, Oregon) with a sampling rate of 500 Hz. Four electrodes placed near the eyes were used to monitor eye movements, while electrodes positioned over the masseter muscles and near the chin were used to monitor muscle tone.

In addition to the sleep recordings, a six-minute period of quiet wakefulness with eyes closed was recorded in the evening prior to the sleep sessions, and was used as a wakefulness reference (Figures 1, 2, S2, S3, and S6).

### Method details

#### Sleep scoring and signal extraction

The EEG signal was offline band-pass filtered between 0.5 and 45 Hz, and sleep staging was performed in 30 second epochs according to standard criteria.

Spontaneous awakenings lasting more than 30 seconds and accompanied by clear-cut EEG wake patterns and movement activity (on EMG channels) were identified by an expert scorer (JC) in the baseline recording. Movement onset was defined as the first detection of movement, either via EMG or on video, whichever occurred first. For spontaneous awakenings, 60 seconds of EEG before and after movement onset were extracted for analysis. For awakenings induced by the alarm sound, 60 seconds of EEG before and after alarm onset were extracted for analysis.

A total of 111 spontaneous awakenings (5.8 ± 1.4 awakenings/night, range: 3-8) were extracted from the baseline recordings, of which 74 occurred during NREM sleep (4.1 ± 1.5/night, range: 2-6; 55 N2 and 19 N3) and 37 during REM sleep (2.3 ± 1.0/night, range: 1 - 4).

From the serial awakening nights, 572 provoked awakenings (13.9 ± 3.2/night, range: 8-22) were extracted, including 438 occurred during NREM sleep (10.7 ± 2.4/night, range: 6-17; 238 N2 and 200 N3), and 134 during REM sleep (3.3 ± 1.2/ night, range: 1-7). The sleep preceding these alarm-induced awakenings was analyzed in a previously published study as part of a control group.^21^

Spontaneous arousals were defined as an abrupt shift in EEG towards higher-frequencies, lasting at least 3 seconds and less than 15 seconds, according to the current criteria of the American Academy of Sleep Medicine (AASM).^32^ Arousals were marked using a full 10-20 montage, with its onset identified on the channel where the shift was first observed. Only spontaneous NREM arousals were extracted for analysis. To isolate arousals without movement, those accompanied by EMG activation were excluded. To do so, the signal in electrodes on the neck and cheek were filtered between 100 and 250 Hz, the envelope of the signal was computed and trials where the envelope exceeded one standard deviation of activity 30 to 20 seconds before arousal onset were rejected. This method was used in order to avoid any false negatives at the price of some false positives. Ultimately, 390 arousals without EMG activation were analyzed (26 ± 15.8/night, range:6-67).

#### Sleep scoring and EEG preprocessing

Channels containing artefactual activity were visually identified and replaced by interpolation based on the remaining channels, using spherical splines (NetStation, Electrical Geodesic Inc.). Arousal data did not undergo any further artefact removal procedure. As for spontaneous and induced awakenings, since they are often associated with important movement artifacts, a more thorough artifact removal procedure was used. This involved the use of Independent Component Analysis (ICA) and Artifact Subspace Reconstruction (ASR) with EEGLAB routines. ICA was used to eliminate ocular, muscular, and cardiac artefacts.^90^ Details on ASR were described in a previous publication.^20^ Briefly, ASR is an adaptive spatial filtering algorithm which rejects artefactual EEG segments based on their deviation from a clean EEG calibration signal using principal component analysis. The ASR procedure was adapted for our analyses, based on visual inspection, in order to maximally remove noise and conserve sleep slow waves.^91^ All subsequent EEG analyses were performed on average-referenced data.

#### EEG analysis

##### Fast Fourier Transform

Fast Fourier transform was applied to non-overlapping 1s epochs to compute signal power in standard frequency bands: delta (1–4 Hz), theta (5–7 Hz), alpha (8–11.5 Hz), sigma (12–16 Hz), beta (18–29.5 Hz). This analysis was also performed on the six-minute quiet wakefulness recordings with eyes closed. For Figure S3A, the second-by-second time course of each frequency band was normalized relative to the wake reference. For Figures 1, 2, S2, and S6A, power was further averaged within subjects, and the median values across subjects were plotted. Paired t-tests comparing subject means at each time point to background sleep and quiet wakefulness was performed. Furthermore, for each individual trial, delta and beta power peaks were identified as maximum values exceeding two standard deviations above background activity. Their timing was plotted histograms in Figure S3B.

##### Source localization

Source localization was performed according to a previously described procedure^1^ using the GeoSource software (Electrical Geodesics, Inc., Eugene, Oregon). Individualized electrode geocoordinates were used to construct the forward model. The source space was constrained to 2,447 dipoles distributed over 7×7×7-mm cortical voxels. The inverse matrix was computed using the standardized low resolution brain electromagnetic tomography (sLORETA) method.^92^ A Tikhonov regularization (λ=10^-2^) procedure was applied to account for the variability in the signal-to noise ratio.^92^

##### Peak activity latency

A latency analysis was performed to determine the spatial order of cortical changes during the awakening process. For each frequency band, the timing of the maximum power values (peak activity) within a time window spanning from 10 seconds before to 45 seconds after movement/sound onset was extracted for each voxel. For each individual awakening, voxels were ranked according to their latency, then normalized to a scale from 1 to 10 (with 1 representing the earliest voxels to reach peak activity and 10 the latest). Median maps of these rankings were calculated across subjects (Figures 3 and S6B). For Figure S5, the same procedure was applied, but random windows unrelated to the awakening (from -45 to -30 seconds prior to induced awakening) were used for each trial. In addition, for both Figures 3 and S5, reference maps were generated by computing the median of 19 randomly generated maps – simulating the median of 19 subjects’ maps, if voxel peak latencies were completely random, providing a visual baseline for comparison.

##### Slow wave analysis

EEG sleep slow waves were detected using a previously validated detection algorithm based on zero-crossing detection,^83^^,^^93^ applied to the 60 seconds before and after movement or alarm sound onset. The EEG signal was referenced to the average of the two mastoid electrodes, downsampled to 128 Hz and bandpass filtered (0.5-4Hz; stop-band at 0.1 and 10 Hz) using a Chebyshev Type II filter (MATLAB, Mathworks). Only slow waves with durations between 0.25 and 1s were considered. Detection was performed for each channel separately. For Figure 6, the following slow wave parameters were extracted: slow wave density (number of slow waves per minute), maximum negative peak amplitude, slope 1 (between the first zero crossing and the negative peak), slope 2 (between the negative peak and the second zero crossing), the number of negative peaks within a slow wave. These parameters were extracted and averaged across detected slow waves within the following windows: -45 to -30s; -30 to -5s, -5 to 0s, 0 to 5s, 5 to 30s. A thorough visual inspection of detected slow waves was performed to identify K-complexes and verify the amplitude-based separation.

**Table:** 

Figure	Analysis	Statistics
Figures 1, 2, and S2; Spontaneous	Time-frequency of fast Fourier transform	Median across subjects of logged z-score on time
Figures 1, 2, and S2; Spontaneous	Fast Fourier transform normalized on wake	Paired t-test vs sleep (-45 to -30s); Paired t-test vs wake eyes closed
Figure S1; Spontaneous	Fast Fourier transform topography	Average (-30 to -5; -5 to 0; 0 to 15)
Figure S3A; Spontaneous	Fast Fourier transform	Z-score over time
Figure S3B; Spontaneous	Peak activity in delta and beta power	
Figure S3C	Pre-processed EEG signal, Fast Fourier transform time frequency and band power	
Figure S4; Spontaneous	Fast Fourier transform topography	Paired t-test vs sleep (-45 to -30s); Paired t-test vs wake eyes closed
Figures 3 and S5; Spontaneous	Peak activity latency maps on source	Median of ranked voxels across subjects
Figure 4; Provoked	Subjective sleepiness scores	Linear mixed models predicting sleepiness based on stage of awakening
Figure 5; Provoked	Fast Fourier transform on source	Linear mixed models predicting sleepiness
Figure 6A; Spontaneous	Slow wave analysis	Paired t-test vs sleep (-45 to -30s)
Figure 6B; Provoked	Slow wave detection	Linear mixed models predicting sleepiness
Figure S6A; Provoked	Time-frequency of fast Fourier transform	Median across subjects of logged z-score on time
Figure S6A; Provoked	Fast Fourier transform normalized on wake	Paired t-test vs sleep (-45 to -30s); Paired t-test vs wake eyes closed
Figure S6B; Provoked	Peak activity latency maps on source	Median of ranked voxels across subjects

### Quantification and statistical analysis

Statistical analyses were performed in MATLAB (version R2021a) and RStudio (version 1.1.463).

#### Student’s t.test

Timepoint by timepoint paired Student’s t-tests were applied to compare power (Figures 1, 2, 6A, and S2) and slow wave parameters (Figure 6A) between the awakening period and the sleep- (-45 to- 30s) and wake baselines. P-values were corrected for multiple comparisons (frequency bands) using a cluster- and probability-based correction.

#### Linear mixed models

To evaluate the relationship between cortical activity and subjective sleepiness we used linear mixed models. Models were applied for each voxel (at the source level) to predict subjective sleepiness based on absolute power spectral density in different frequency bands (Figure 5) and different slow wave parameters (Figure 6B). Subject identity and time of night were included as a random factors in all analyses. As between-subject variability was accounted for by including subject identity as a random factor, all EEG power spectral density models were performed on absolute (non-normalized) power values. For each model, we obtained the Wald statistics, which is the squared ratio of the fixed factor estimate over its standard error. To account for multiple comparisons, a cluster- and probability-based correction was used. In order to create a dummy population, labels corresponding to the dependent variables were shuffled within subjects 1000 times. For each permutation, the model was applied and neighboring electrodes with p-values less than 0.05 were identified as a cluster. A cluster statistics was produced by summing Wald statistics inside each significant cluster and by dividing by the number of electrodes in the cluster, resulting in one value for each identified cluster. For each permutation, only the absolute maximal value of all cluster statistics was retained. A threshold for significance was set at the 95th percentile of the dummy cluster statistics distribution. The cluster statistics obtained in the real dataset were considered significant if above this threshold. This procedure was applied for each electrode/voxel.

## Supplemental information

Document S1. Figures S1–S6

Table S1. Cortical maps of total power and absolute latencies, related to Figure 3 Table listing median absolute latencies of peak activity for each voxel – the Brodmann area associated with each voxel is reported for reference.

Document S2. Article plus supplemental information
