# Supplementary material for: Cortical activity upon awakening from sleep reveals consistent spatio-temporal gradients across sleep stages in human EEG
Source: Curr Biol. 2025 Aug 18;35(16):3812–3824.e3. doi: 10.1016/j.cub.2025.06.064 (PMC12459234; doi:10.1016/j.cub.2025.06.064)
Supplement: Document S1. Figures S1–S6 [file mmc1.pdf]

Current Biology, Volume *35*

Supplemental Information

Cortical activity upon awakening from sleep reveals consistent spatio-temporal gradients across sleep stages in human EEG

Aurélie M. Stephan, Jacinthe Cataldi, Amrita Singh Virk, and Francesca Siclari

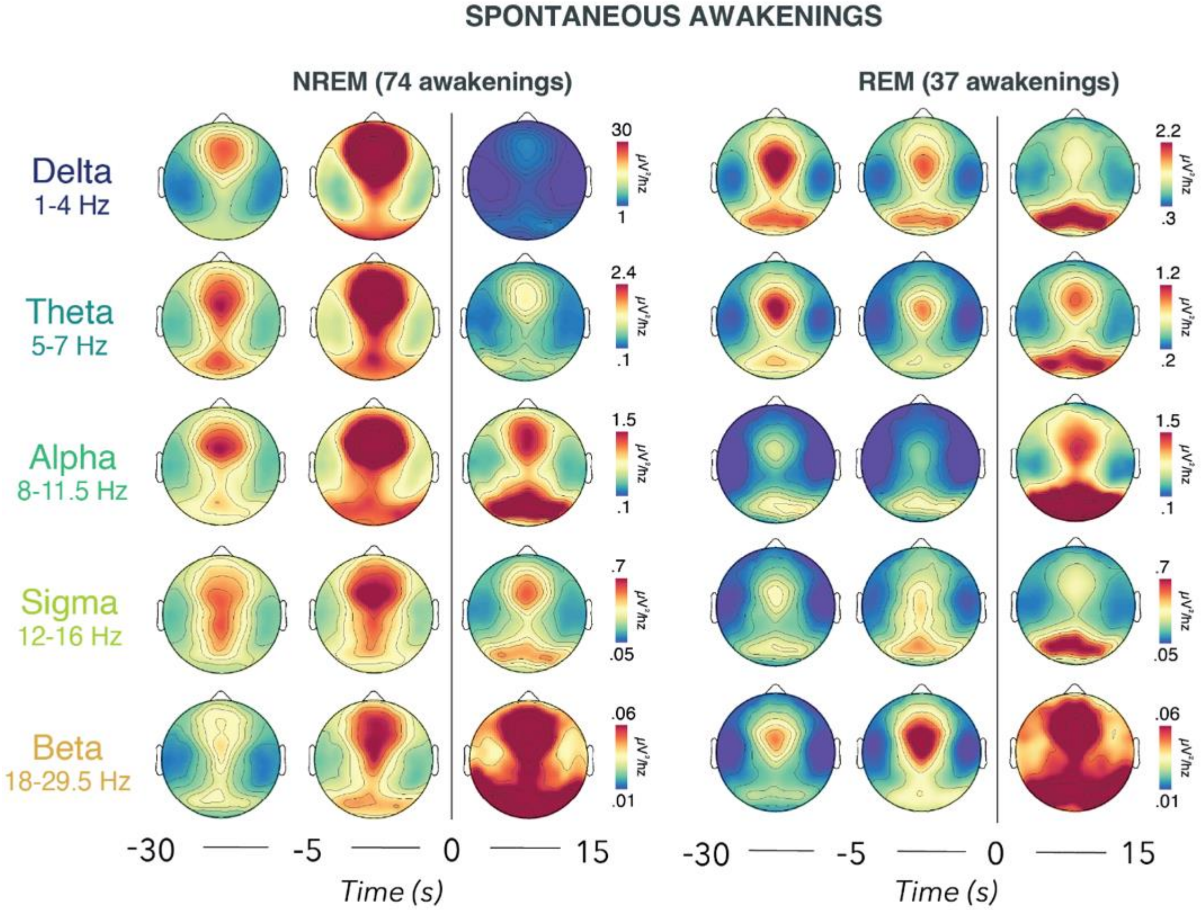

Figure S1. EEG scalp topography timecourse upon spontaneous awakenings out of NREM and REM sleep, Related to Figure 1. Togopraphical representation of average fast Fourier transform in six frequency bands of interest in NREM **(left, N=19 participantes)** and REM sleep **(right, N=17 participants)** in three time windows: background sleep *(-30 to -5 seconds)*, pre-awakening sleep *(-5 to 0)* and awakening *(0 to 15 seconds after movement onset)*. Amplitude scales are fixed across time windows but different for each frequency band and sleep stage to allow the assessment of change in amplitude.

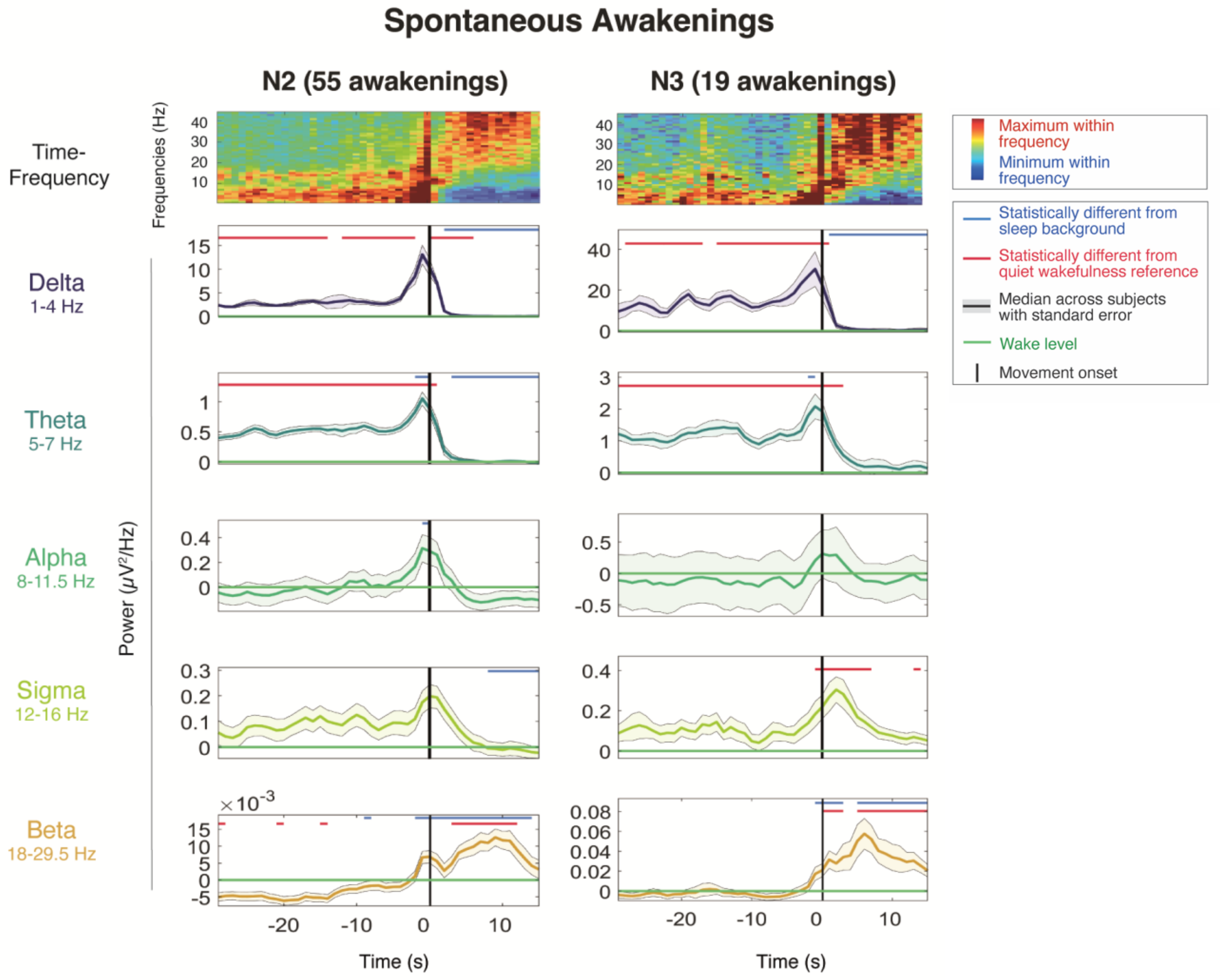

Figure S2. Timecourse of EEG activity of spontaneous awakenings out of NREM-2 and NREM-3 sleep, Related to Figure 1. Timecourse of fast Fourier transform across frequencies **(first row)** and separated into six frequency bands of interest **(second to bottom row)** averaged across 185 innermost channels in NREM2 sleep **(left, N=18 participants)** and NREM3s sleep **(right, N=12 partifcipants)**. The time-frequency plots **(first row)** are logged and z-scored across time for each subject, and the median across subjects is plotted. The frequency band plots **(second to bottom row)** are normalized on resting wakefulness for each subject, the median across subjects is plotted in bold and the standard error as an area around it. The black line at 0 denotes movement onset. The green horizontal line displays the wake level reference. On the top of each frequency band plot, the blue line indicates the timepoints at which the power is significantly different from the background sleep reference (-45 to -30s) and the red line indicates the timepoints at which the power is significantly different from the quiet wake reference (evening prior) .

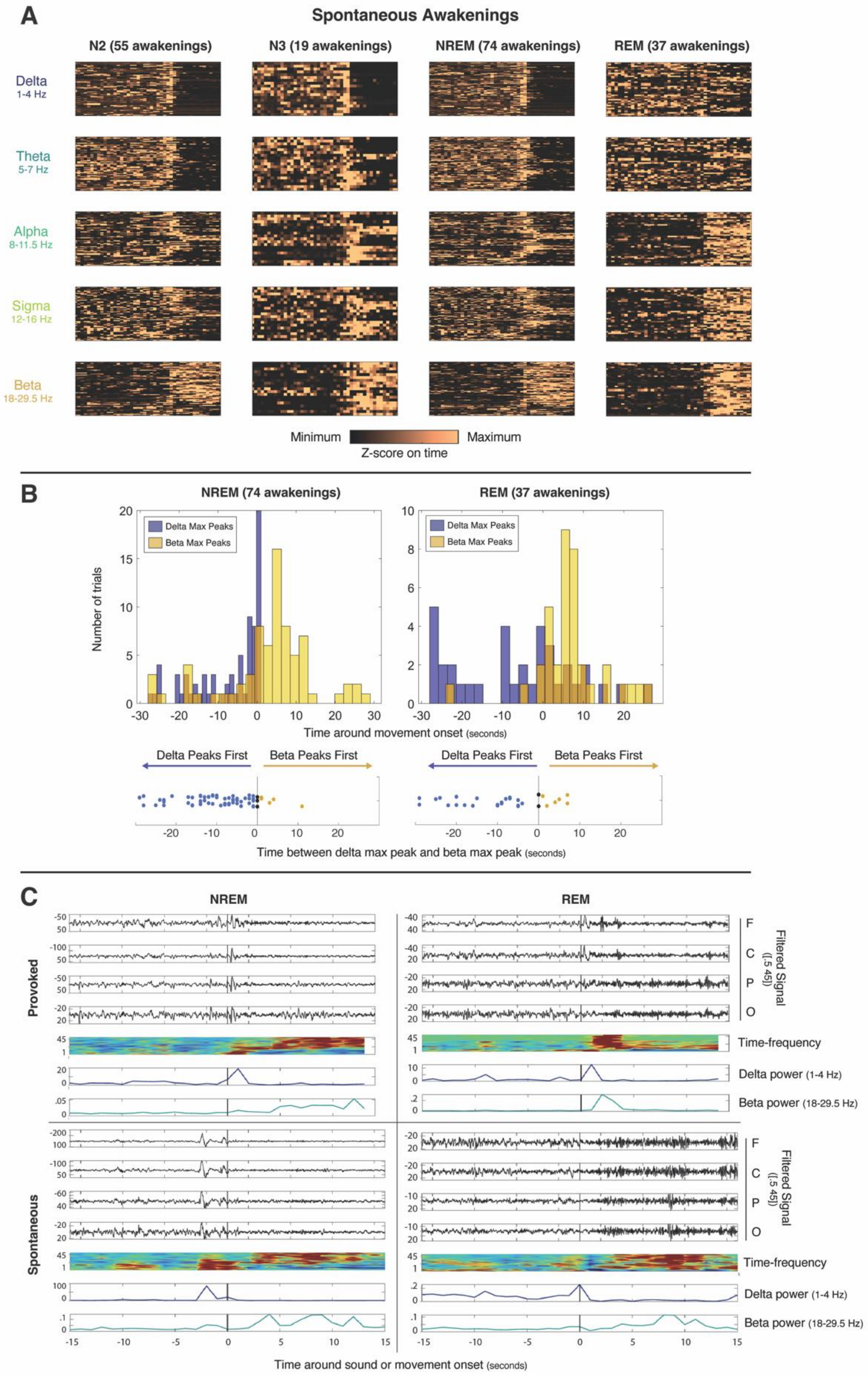

Figure S3. Time course, low- and high-frequency peak order and illustrative examples of individual trials, Related to Figure 1. Raster plots of timecourse for power in frequency bands of interest, displayed for spontaneous awakenings out of N2 (N=18), N3 (N=12), NREM (N=19) and REM sleep (N=17) **(A)** and distribution of individual delta and beta peak time and order **(B)**. **(A)** For each frequency band, the raster plot displays individual trials on the y axis and time on the x axis. The spectral power values have been logged and z-scored and the color scale indicates the within-trial maximum values in light copper color. **(B)** The histograms display the peak time of delta power (blue) and beta power (yellow) in all spontaneous awakenings from NREM (left) and REM sleep (right). Zero corresponds to the movement onset. Underneath the histogram, a scatter plot indicates for each awakening, the delay between the delta and beta peak. Negative values (blue dots) indicate trials in which delta peaked before beta. Positive values (yellow dots) indicate trials in which beta peaked before delta. **(C)** EEG signal from midline frontal, central, parietal and occipital midline channel are plotted. The signal is filtered and post-ICA, referenced to linked mastoid (row 1-4). Timecourse of fast Fourier transform averaged across innermost 185 channels (row 5). The time-frequency plot is logged and z-scored on the time window for each awakening. The two bottom row display the timecourse of delta (1-4 Hz) and beta (18-29.5 Hz) power. All plots show the 15 seconds prior and after movement onset.

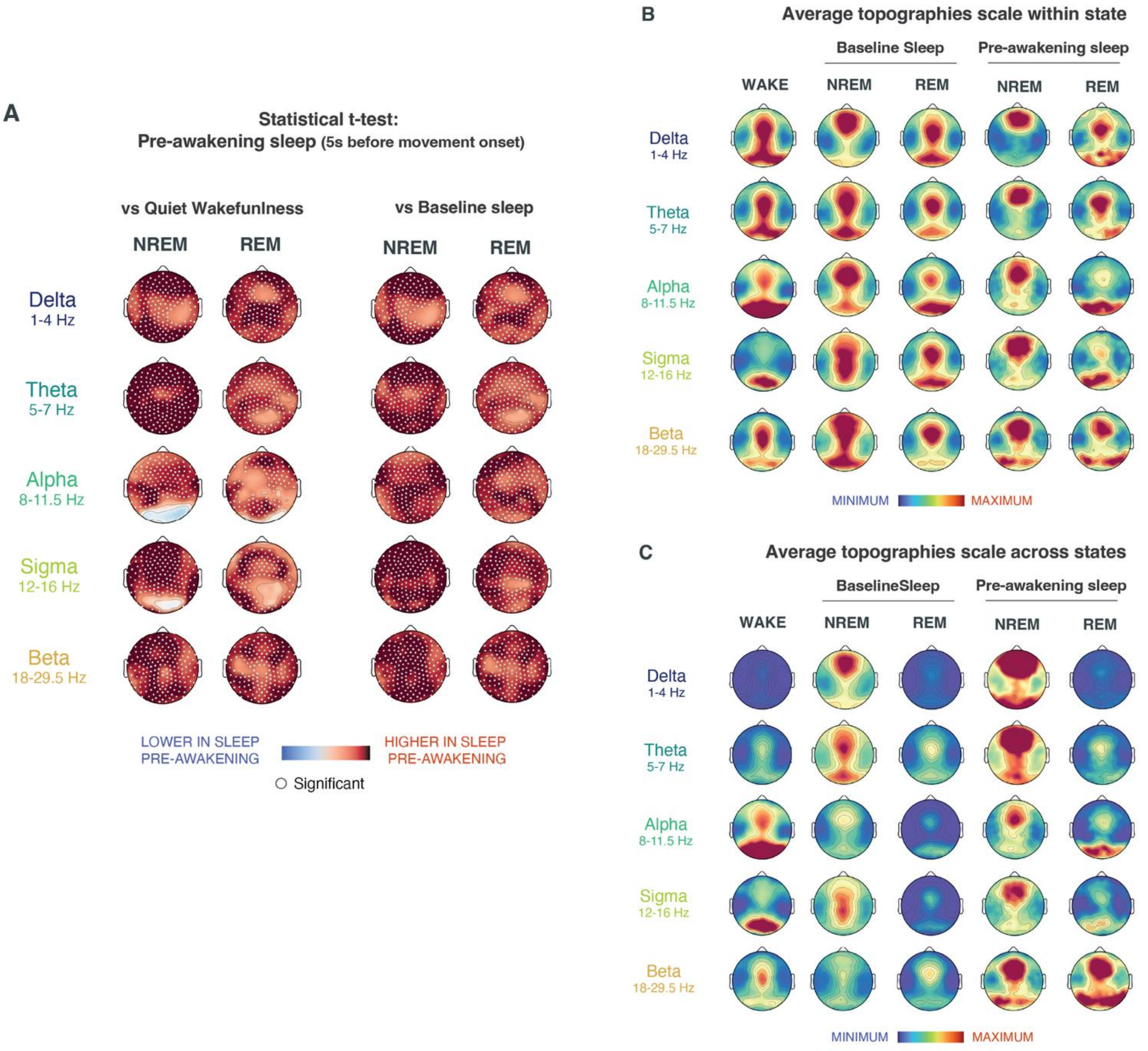

Figure S4. Topography of spectral power peaks, Related to Figure 1 and Figure 3. (A) Topographical representation of t-values for the contrast sleep prior to awakening versus pre-sleep quiet wakefulness **(left)** and versus baseline sleep (45 to 30 sec prior to movement onset, (**right**) in each frequency band of interest (two-tailed paired t-test) in NREM sleep (N=19 participants) and REM sleep (N=17 participants). Red indicates regions where the activity in a spectral band was higher in the sleep prior to awakening (last 5 seconds prior to movement onset) compared to quiet wakefulness or baseline sleep. White dots indicate statistically significant channels. Average topographies in pre-awakening sleep (last 5 seconds prior to movement onset) and baseline sleep (45 to 30 seconds prior to movement onset) are also displayed with a scale within state **(B)** in order to assess the topographies; and with a fixed scale across states **(C)** in order to compare the amplitude between states.

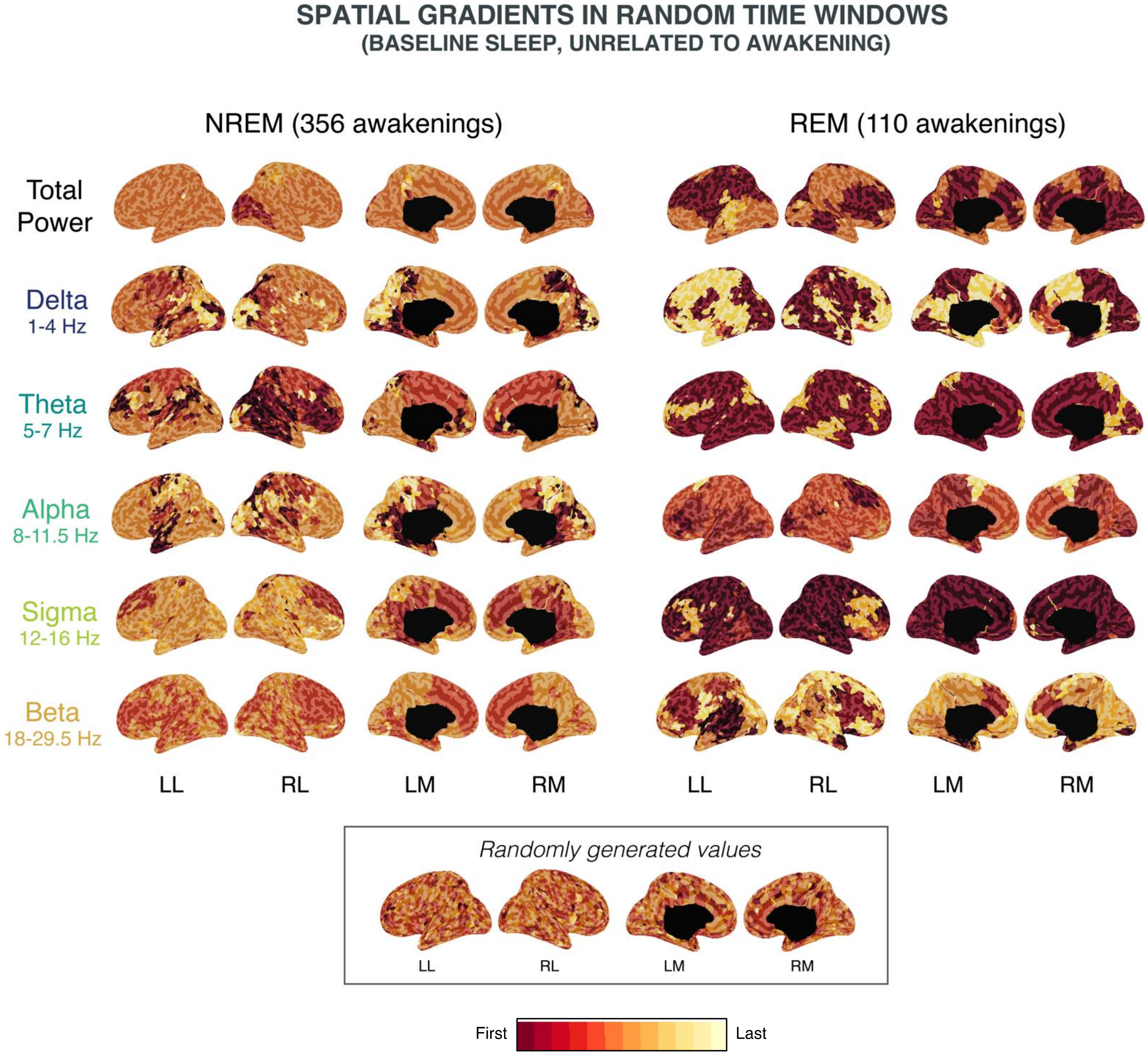

Figure S5. Cortical maps of latency to peak activity locked to background sleep, Related to Figure 3. The timing of peak activity is displayed on source reconstructed data. Latency maps display, for each frequency band, the timing at which regions reach their maximal activity in the window from -30 to -15s prior to the awakening, to visualize the spatial distribution of the peak around an arbitrary timepoint in the background sleep; red indicates regions peaking early and yellow regions peaking late. In the bottom, the cortical map of randomly generated values is displayed for reference. *LL: Left Lateral; LM: Left Medial; RL: Right Lateral; RM: Right Medial.*

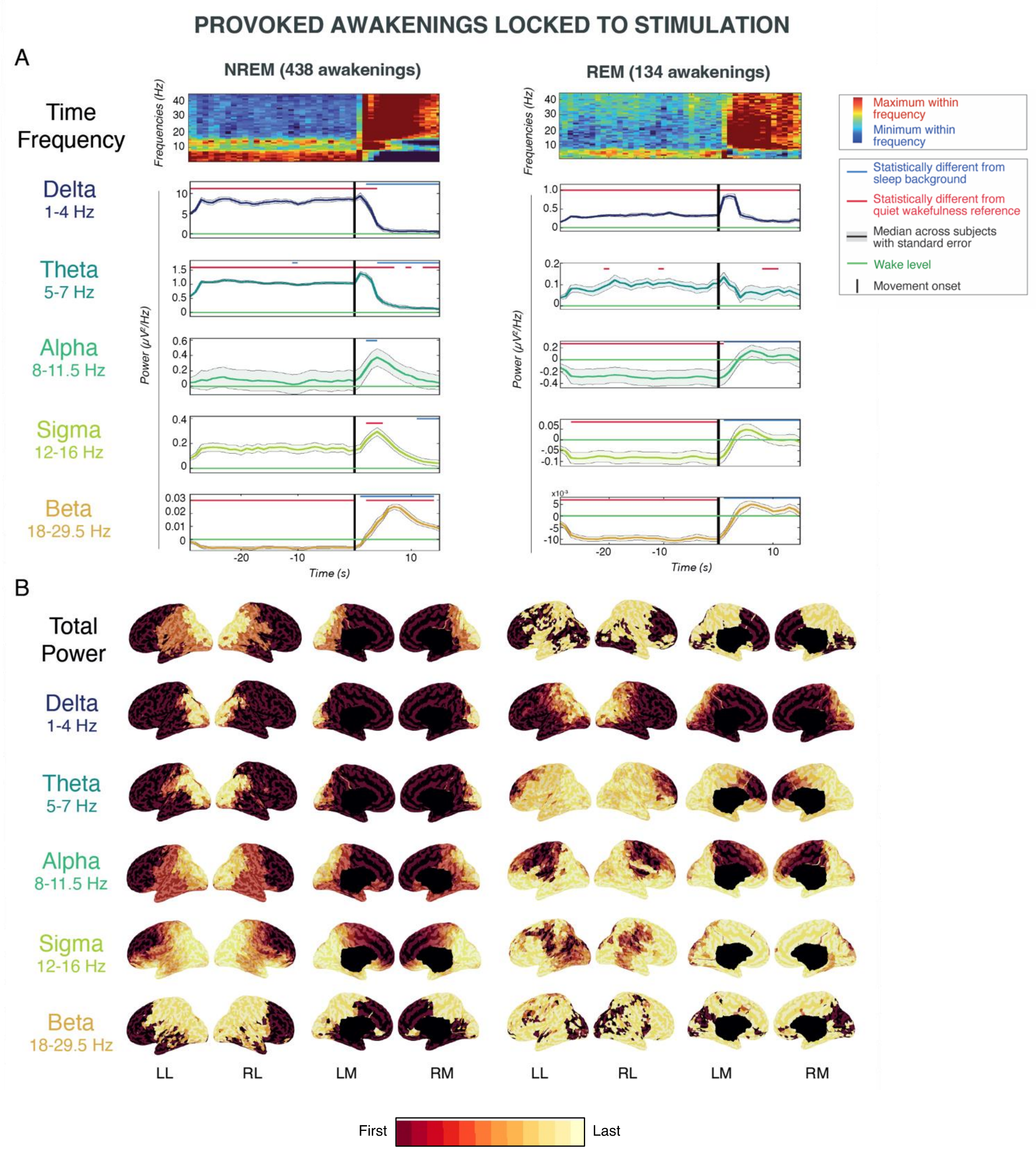

Figure S6. Timecourse of EEG activity and cortical maps of latency to peak activity upon provoked awakenings out of NREM and REM sleep, Related to Figure 5. Timecourse of fast Fourier transform across frequencies **(first row)** and separated into six frequency bands of interest **(second to bottom row)** averaged across 185 innermost channels **(A)** and latency maps in total power and frequency bands of interest **(B)**. **(A)** The time-frequency plots are logged and z-scored across time for each subject and the median across subjects is plotted **(first row)**. The frequency bands plots are normalized on wake for each subject, the median across subjects is plotted in bold and the standard error as an area around it. The black line at 0 denotes movement onset. The green horizontal line at 0 displays the wake level reference. On the top of each frequency band plot, the blue line indicates the timepoints at which the power is significantly different from background sleep reference (-45 to -30) and the red line indicates the timepoints at which the power is significantly different from the quiet wake reference (evening prior). **(B)** The timing of peak activity is displayed on source reconstructed data. Latency maps display, for each frequency band, the timing at which regions reach their maximal activity in the window from -15 to +15, to visualize the spatial repartition of the peak around the awakening behavioral onset; red indicates regions peaking early and yellow regions peaking late. LL: Left Lateral; LM: Left Medial; RL: Right Lateral; RM: Right Medial.
